# Marine medaka PKCα promotes red-spotted grouper nervous necrosis virus entry by orchestrating MYL3-mediated macropinocytosis and cofilin-dependent actin remodeling

**DOI:** 10.1128/jvi.02064-25

**Published:** 2026-02-25

**Authors:** Lan Yao, Xiaogang Yang, Haifeng Li, Xingchen Xiong, Meisheng Yi, Kuntong Jia

**Affiliations:** 1 School of Marine Sciences, Sun Yat-sen University, Guangzhou, China; 2 Guangdong Provincial Key Laboratory of Marine Resources and Coastal Engineering, Guangzhou, China; University of Kentucky College of Medicine, Lexington, Kentucky, USA

**Keywords:** nervous necrosis virus, protein kinase C alpha, cofilin, macropinocytosis

## Abstract

Protein kinase C alpha (PKCα) is a central signaling molecule implicated in various cellular processes, including viral infections. However, its role in fish viruses, particularly nervous necrosis virus (NNV), remains elusive. Here, we report that PKCα from marine medaka (MmPKCα) facilitates red-spotted grouper NNV (RGNNV) entry by bridging viral receptor marine medaka myosin light chain 3 (MmMYL3, a known RGNNV receptor mediating macropinocytosis) binding to downstream actin dynamics. RGNNV infection upregulates MmPKCα expression and activates its phosphorylation. Gain- and loss-of-function studies demonstrated that MmPKCα enhances NNV entry, dependent on its kinase activity. We found that MmPKCα interacts with the RGNNV capsid protein via its C-terminal (CT) domain. Although MmPKCα localized to the cell surface, it did not function as an RGNNV receptor, evidenced by unaltered viral binding, inability to render non-susceptible cells permissive, and lack of inhibition by anti-PKCα antibodies/recombinant MmPKCα protein. Instead, MmPKCα directly interacts with MmMYL3 and is essential for MmMYL3-mediated macropinocytosis. Furthermore, MmPKCα binds to marine medaka cofilin (MmCFL1/2) through its CT domain and suppresses cofilin phosphorylation at Ser3, thereby activating cofilin and promoting actin rearrangement critical for macropinocytosis. Our study uncovers a novel MmMYL3-MmPKCα-cofilin signaling axis that RGNNV exploits to enter host cells, highlighting PKCα as a potential therapeutic target for controlling NNV infection.

**IMPORTANCE:**

Nervous necrosis virus (NNV) causes devastating losses in global aquaculture. Here, we identify a novel MmMYL3-MmPKCα-cofilin signaling axis that NNV exploits to enter host cells via macropinocytosis. This finding advances our understanding of fish virus invasion mechanisms, uncovering how NNV hijacks host signaling for entry. Beyond basic virology insights, our work highlights protein kinase C alpha (PKCα) as a promising therapeutic target, laying the groundwork for developing targeted antivirals to control NNV.

## INTRODUCTION

Viral nervous necrosis (VNN), also known as viral encephalopathy and retinopathy, is a devastating infectious disease affecting over 120 marine and freshwater fish species, including economically critical species. Characterized by severe neuropathological changes and high mortality in larval and juvenile fish, VNN causes substantial economic losses to the global aquaculture industry (1). The etiological agent, nervous necrosis virus (NNV), belonging to the family Nodaviridae, is a non-enveloped, icosahedral virus with a bipartite single-stranded RNA genome (RNA1 and RNA2) (2). RNA1 encodes the RNA-dependent RNA polymerase (RDRP) for viral replication, while RNA2 encodes the capsid protein (CP), the only structural protein of NNV, which is critical for viral attachment and entry into host cells (3).

A critical step in viral pathogenesis is virus entry into host cells, as it determines viral tropism and initiates the infection cycle. Previous studies have revealed that NNV particles utilize receptor-mediated clathrin-mediated endocytosis (CME) and macropinocytosis to invade host cells (4, 5). Among these, macropinocytosis is a specialized, actin-dependent endocytic process characterized by extensive plasma membrane ruffling, formation of large vesicles (macropinosomes), and independence from clathrin or caveolin (6). Unlike CME selective for small cargo, macropinocytosis enables efficient internalization of large particles such as viruses, and its activation by pathogens is tightly regulated by a network of signaling molecules, including cell surface receptors, small GTPases, and serine/threonine kinases (7). Elucidating the molecular regulators of red-spotted grouper NNV (RGNNV)-induced macropinocytosis is therefore critical for understanding NNV pathogenesis and developing targeted antiviral strategies.

The protein kinase C (PKC) family, comprising 12 serine/threonine kinases divided into classical (α, βI, βII, γ), novel (δ, ε, θ, η), and atypical (ζ, ι/λ) subtypes, emerges as a key player in regulating endocytic processes and viral infections (8, 9). In mammalian viral infections, PKC activation is important in the early stages of respiratory syncytial virus and herpes simplex virus type 1 infection (10, 11). PKCα and PKCθ are also intimately associated with viral replication of human immunodeficiency virus and chikungunya virus (12, 13). Apart from these, PKCs could also serve as a crucial regulatory factor in toll-like receptor, AKT/mTOR, NF-κB, and other essential signaling pathways triggered by viral infection, further highlighting their central role in host–virus interactions (14–16). Despite this extensive evidence in mammalian systems, the function of PKCs, particularly the evolutionarily conserved classical subtype PKCα, in fish viral infections remains largely uncharacterized.

A defining feature of macropinocytosis is actin cytoskeleton rearrangement, which drives membrane ruffling and macropinosome formation (17). The key regulator of actin dynamics is cofilin, a member of the actin-depolymerizing factor/cofilin family that severs and depolymerizes filamentous actin to maintain actin turnover. Cofilin activity is tightly controlled by phosphorylation at serine 3 (Ser3): phosphorylation inactivates cofilin, while dephosphorylation restores its actin-severing activity (18). Numerous pathogens, including viruses, promote their entry into host cells by stimulating actin cytoskeleton remodeling (19). For example, in viral infections, cofilin-dependent actin remodeling is required for the entry of influenza A virus, rabies virus, and porcine hemagglutinating encephalomyelitis virus (20–23). Notably, a PKCδ-cofilin signaling pathway was recently shown to mediate macropinocytosis of porcine reproductive and respiratory syndrome virus (24), suggesting a conserved regulatory module for virus-induced actin remodeling. However, whether the PKC-cofilin signaling pathway regulates RGNNV-induced macropinocytosis remains unknown.

We previously identified marine medaka (*Oryzias melastigma*) myosin light chain 3 (MmMYL3) as a cell surface receptor for RGNNV and demonstrated that MmMYL3 mediates RGNNV entry exclusively via macropinocytosis (4). However, which downstream signaling molecules transduce MmMYL3-dependent signals to drive macropinocytosis and whether PKCα, an established regulator of endocytosis and actin dynamics, participates in this process remains still unknown. The present study investigated the regulatory role of marine medaka PKCα (MmPKCα) in RGNNV infection, clarified MmPKCα’s modulation of RGNNV-induced macropinocytosis, and its dependence on the MmMYL3 receptor. Our results uncover a novel MmMYL3-MmPKCα-cofilin signaling axis exploited by RGNNV for host cell entry, providing new insights into NNV pathogenesis and identifying MmPKCα as a potential therapeutic target for VNN control.

## RESULTS

### MmPKCα is activated by RGNNV and promotes viral entry

**Fig 1:**
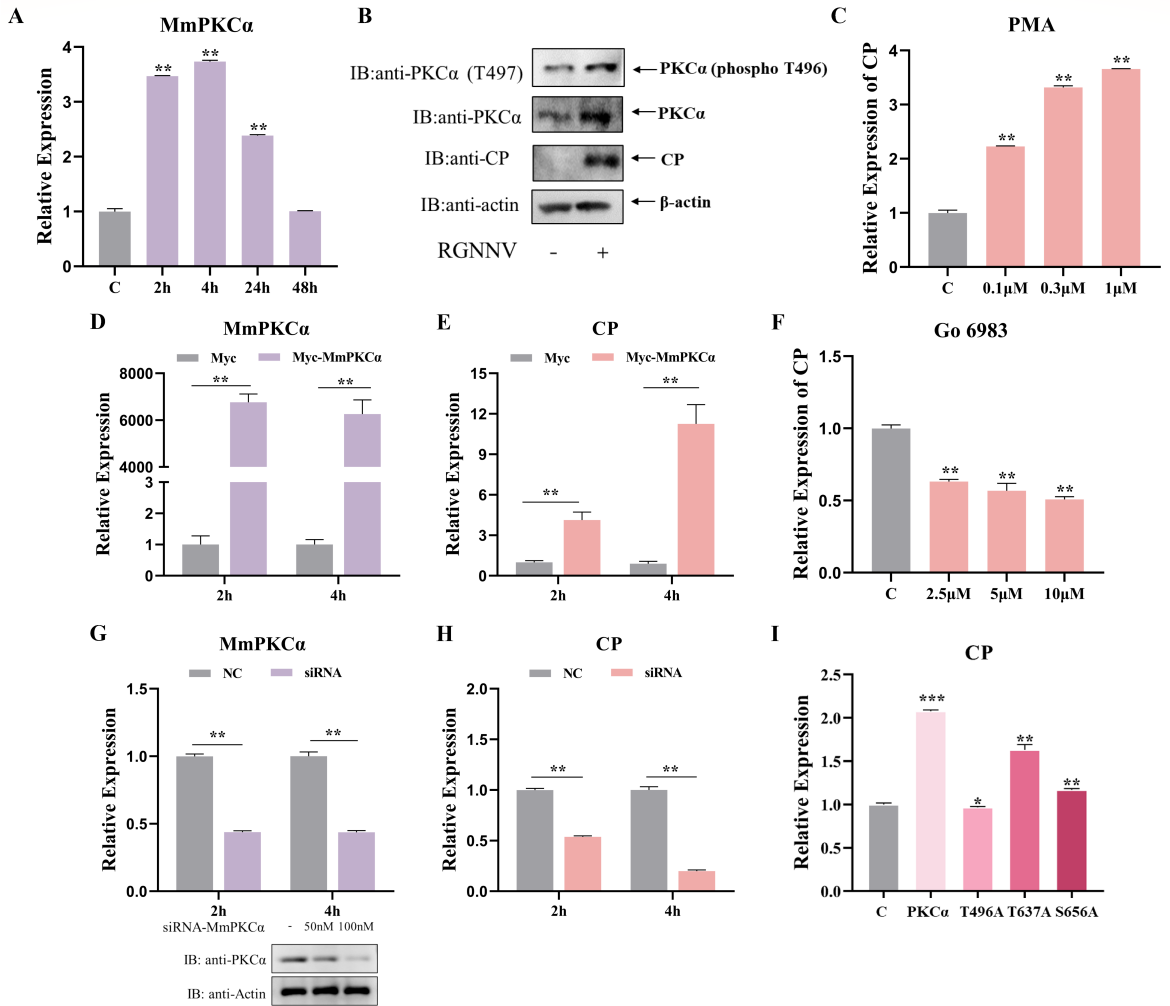
MmPKCα promotes RGNNV infection. (**A**) hMMES1 cells were infected with RGNNV (multiplicity of infection [MOI] = 1) for 2, 4, 24, and 48 h, respectively. Then, the cells were lysed for qRT-PCR to detect *MmPKCα* mRNA expression. (**B**) hMMES1 cells were infected with RGNNV (MOI = 1) for 24 h and then were subjected to immunoblot assays using anti-PKCα or anti-PKCα (phosphor T497) abs. (**C**) hMMES1 cells were treated with PKC activator phorbol 12-myristate 13-acetate (PMA) for 4 h, then infected with RGNNV (MOI = 1) for 4 h at 28°C, and the cells were lysed for qRT-PCR to detect the expression of *CP*. (**D and E**) hMMES1 cells were transfected with MmPKCα-Flag or Flag plasmid (control) and infected with RGNNV (MOI = 1) for 2 and 4 h, respectively. Then, the cells were lysed for qRT-PCR to detect the expression of *MmPKCα* and *CP*. (**F**) qRT-PCR analysis of CP expression in PKC inhibitor Go 6983 treated hMMES1 cells infected with RGNNV (MOI =1) for 4 h. (**G–H**) qRT-PCR analysis of *MmPKCα* and *CP* expression in siMmPKCα- or control-transfected hMMES1 cells infected with RGNNV (MOI = 1) for 2 and 4 h. (**I**) hMMES1 cells were transfected with MmPKCα-Flag or its point mutants of T496, T637, or S656 plasmids and infected with RGNNV (MOI = 1) for 4 h, respectively. Then, the cells were lysed for qRT-PCR to detect the expression of *CP*. The results are presented as mean ± SD. Statistical significance was determined by an unpaired two-tailed Student’s t test. *P < 0.05, **P < 0.01, ***P < 0.001. Data are representative of three independent experiments.

To investigate the potential involvement of MmPKCα in RGNNV infection, we first analyzed MmPKCα expression dynamics in RGNNV-infected hMMES1 cells. Quantitative reverse transcription-PCR (qRT-PCR) analysis revealed a significant upregulation of *MmPKCα* mRNA in hMMES1 cells following RGNNV infection, with a peak at 4 h post-infection (hpi) (Fig. 1A), suggesting that MmPKCα might be involved in the early stages of infection. Western blot further confirmed RGNNV infection increased total MmPKCα protein levels and specifically enhanced its phosphorylation at Thr496 (a key activation loop residue) (Fig. 1B), indicating RGNNV induces MmPKCα activation.

We next performed functional studies to determine if MmPKCα activity influences viral entry. Treatment with the PKC activator phorbol 12-myristate 13-acetate (PMA) or ectopic expression of MmPKCα significantly increased the levels of RGNNV *CP* mRNA at 2 and 4 hpi (Fig. 1C through E). Conversely, pharmacological inhibition of PKC with Go 6983 or small interfering RNA (siRNA)-mediated knockdown of MmPKCα markedly reduced viral *CP* mRNA levels (Fig. 1F through H). To confirm the importance of MmPKCα kinase activity, we generated MmPKCα phosphorylation-deficient mutants (T496A, T637A, S656A). Every single mutation impaired the ability of MmPKCα to enhance CP expression (Fig. 1I). Together, these results demonstrate that RGNNV activates MmPKCα, and MmPKCα promotes RGNNV entry in a kinase activity-dependent manner.

### MmPKCα interacts with RGNNV CP via its CT domain

**Fig 2:**
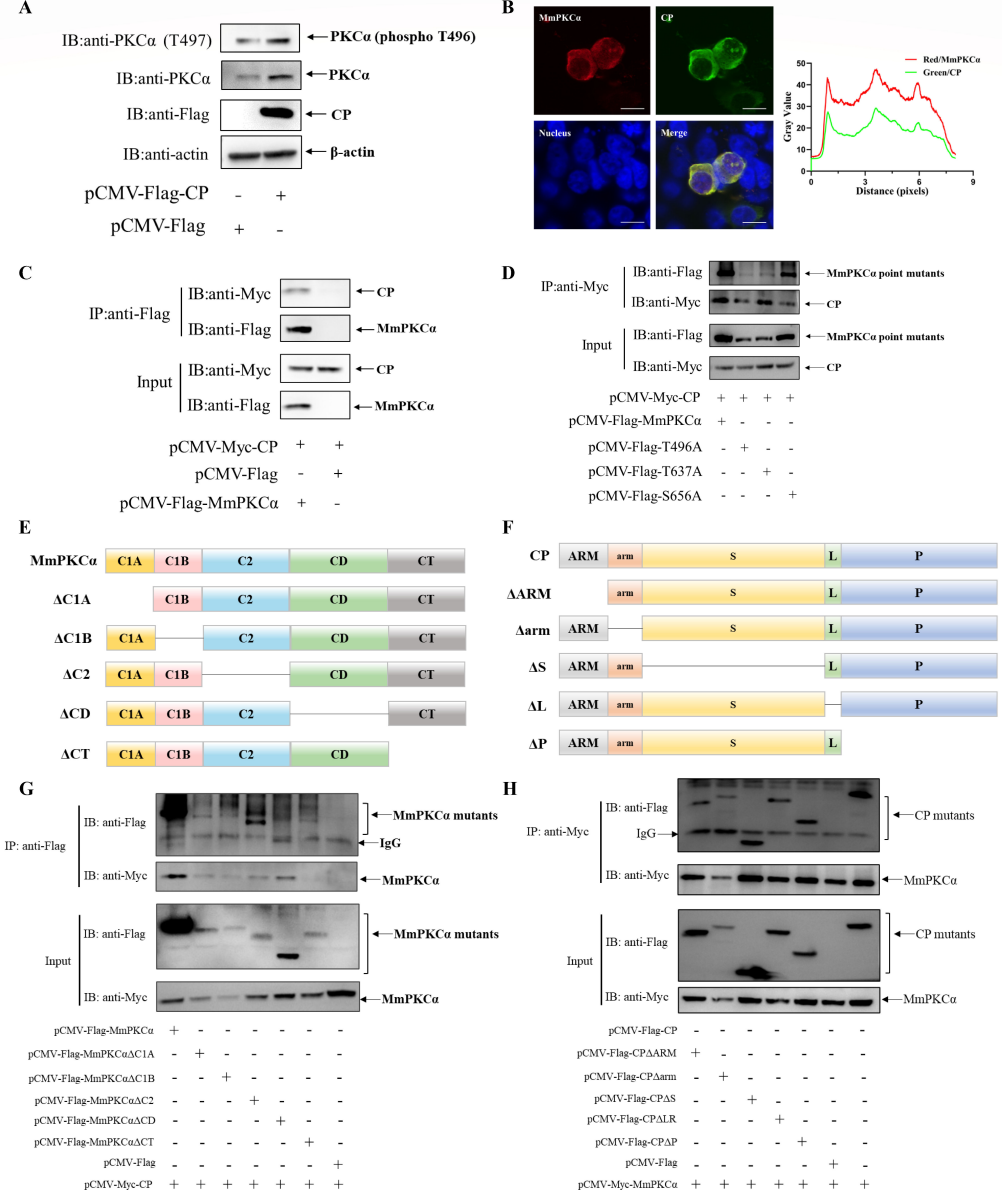
MmPKCα interacts with CP. (**A**) hMMES1 cells were transfected with CP-Flag or Flag plasmids for 24 h and then were subjected to immunoblot assays using anti-PKCα or anti-PKCα (phosphor T497) abs. (**B**) HEK293T cells were co-transfected with MmPKCα-Flag and CP-Myc plasmids. MmPKCα (red) and CP (green) were detected by immunofluorescence staining with anti-Flag or anti-Myc abs, respectively. Nucleus was stained by 4′,6-diamidino-2-phenylindole (DAPI), bar = 10 µm. (**C**) Immunoprecipitation (IP) (with anti-Flag abs) and immunoblot analysis (with anti-Flag and anti-Myc abs) of HEK293T cells transfected with plasmids encoding MmPKCα-Flag and CP-Myc for 48 h. (**D**) IP (with anti-Flag abs) and immunoblot analysis (with anti-Flag and anti-Myc abs) of HEK293T cells transfected with plasmids encoding MmPKCα point mutants-Flag and CP-Myc for 48 h. (**E**) Schematic diagram of MmPKCα and its truncated mutants. (**F**) Schematic diagram of CP and its truncated mutants. (**G**) HEK293T cells were co-transfected with CP-Myc and different Flag-tagged MmPKCα mutants for 48 h. Co-IP assays were performed as described above. (**H**) IP (with anti-Myc abs) and immunoblot analysis (with anti-Flag and anti-Myc abs) of HEK293T cells transfected with plasmids encoding MmPKCα-Myc and Flag-tagged full-length CP or its truncated mutants for 48 h.

Having established that MmPKCα promotes RGNNV entry, we sought to understand the molecular basis of this proviral function. Given that CP is the sole viral structural protein and mediates virus entry, we investigated a potential interplay between MmPKCα and CP. Strikingly, CP overexpression alone was sufficient to elevate both MmPKCα protein levels and its phosphorylation at Thr496 (Fig. 2A), indicating that RGNNV might activate MmPKCα via its CP. Immunofluorescence (IF) showed that MmPKCα and CP proteins colocalized in the cytoplasm of HEK293T cells (Fig. 2B). Co-immunoprecipitation (Co-IP) confirmed reciprocal binding between MmPKCα and CP proteins (Fig. 2C). Furthermore, we found that their interaction was not dependent on MmPKCα’s phosphorylation status, as the phospho-deficient triple mutants (T496A/T637A/S656A) still bound CP (Fig. 2D).

To map the interaction interfaces, we constructed a series of truncation mutants for both MmPKCα and CP (Fig. 2E and F). Co-IP experiments revealed that the C-terminal (CT) domain of MmPKCα was indispensable for binding to CP, as its deletion (ΔCT) completely abolished the interaction, while other domain deletions had no effect (Fig. 2G). In contrast, no single domain deletion in CP disrupted its binding to MmPKCα (Fig. 2H), suggesting that CP engages MmPKCα through a multi-site or conformational interface.

### MmPKCα is not an RGNNV receptor

**Fig 3:**
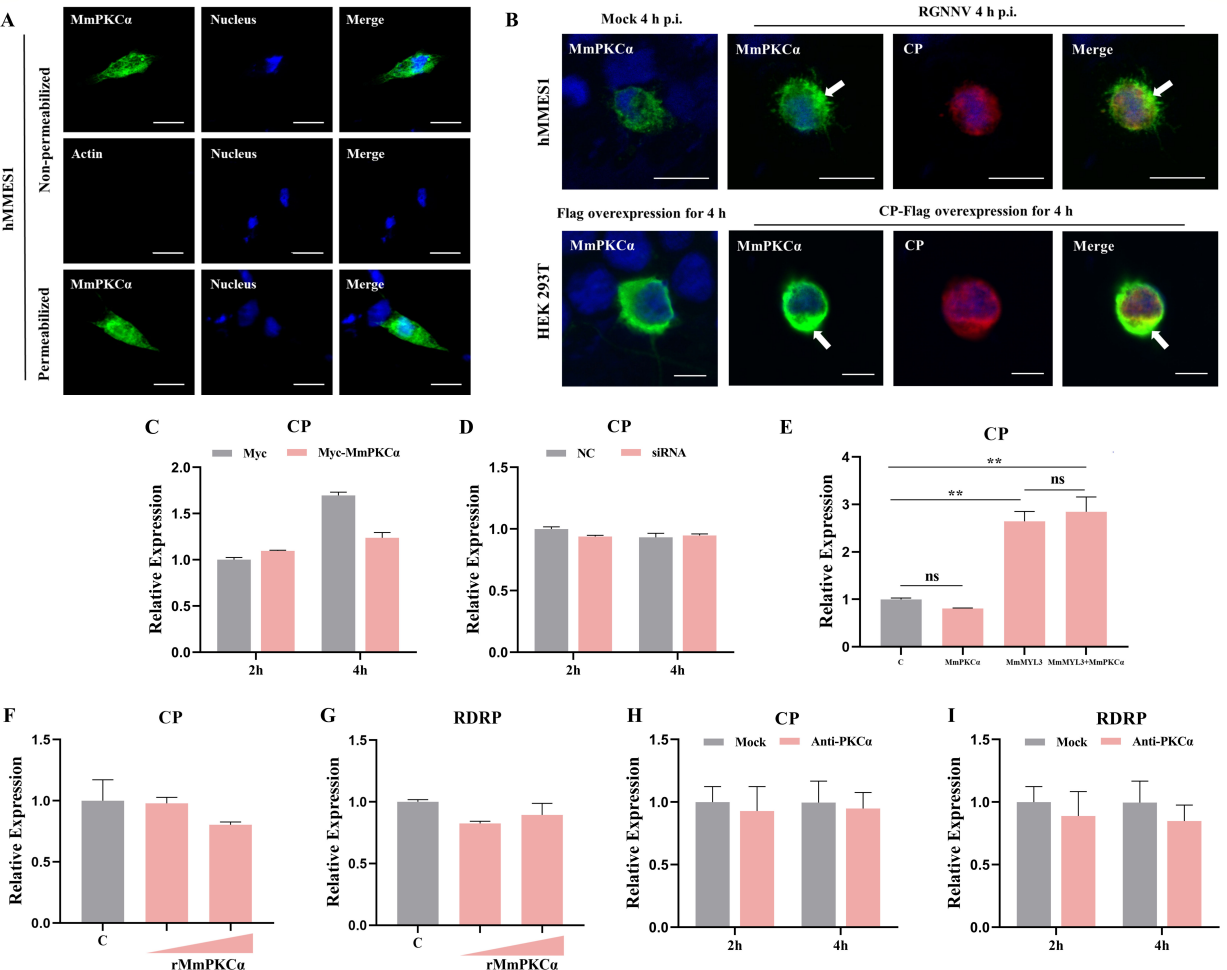
MmPKCα is not the receptor of RGNNV. (**A**) hMMES1 cells transfected with MmPKCα-Myc plasmids were fixed with formalin, treated with Triton X-100 or not, and immunostained with anti-Myc or anti-Actin abs, respectively. Cell nuclei were stained with DAPI, bar = 10 µm. (**B**) hMMES1 cells were infected with RGNNV (MOI = 1) for 4 h, HEK 293T cells were transfected with MmPKCα-Myc and CP-Flag plasmids for 24 h, cells were then fixed and nuclei counterstained with DAPI and imaged on a confocal microscope. Bar = 10 µm. (**C**) hMMES1 cells were transfected with MmPKCα-Flag or Flag plasmids for 24 h, respectively. Then, cells were infected with RGNNV (MOI = 1) at 4°C for 4 h, and the expression of CP was quantified by qRT-PCR. (**D**) qRT-PCR analysis of CP expression in siMmPKCα- or control-transfected hMMES1 cells infected with RGNNV at 4°C for 4 h. (**E**) HEK293T cells were transfected with MmPKCα-Myc, MmMYL3-Myc, MmPKCα-Myc+MmMYL3-Myc, or Myc plasmids, respectively. Then, transfected cells were infected with RGNNV (MOI = 5) for 24 h. Next, the cells were washed to remove any unbound viruses, and total RNA was extracted for CP detection by qRT-PCR. (**F and G**) RGNNV was incubated with purified MmPKCα-GST proteins (100 or 500 ng) for 4 h at 4°C, then was added to hMMES1 cells, which were further incubated for 4 h at 4°C. Cells were washed with PBS three times and harvested for CP (**F**) and RDRP (**G**) expression detection. (**H and I**) hMMES1 cells were incubated with commercial anti-human PKCα abs (1:50) for 4 h and then infected with RGNNV for 2 or 4 h at 4°C. After being washed with PBS three times, cells were harvested for *CP* (**H**) and *RDRP* (**I**) expression detection.The results are presented as mean ± SD. Statistical significance was determined by an unpaired two-tailed Student’s t test. ns, not significant, **P < 0.01. Data are representative of three independent experiments.

The robust interaction between MmPKCα and CP prompted us to investigate whether MmPKCα itself serves as a cell surface receptor for RGNNV. IF analysis of non-permeabilized cells confirmed that a portion of MmPKCα localizes to the plasma membrane (Fig. 3A), and this surface-associated pool increased upon RGNNV infection or CP overexpression (Fig. 3B). However, functional assays argued against the receptor role of MmPKCα. First, neither overexpression nor knockdown of MmPKCα affected the binding of RGNNV virions to hMMES1 cells at 4°C (Fig. 3C and D). Second, unlike the known receptor MmMYL3, MmPKCα overexpression failed to render refractory HEK293T cells permissive to RGNNV entry. Similarly, the inclusion of MmPKCα did not significantly affect the enhancing effect of MmMYL3 (Fig. 3E). Third, pre-incubating the virus with recombinant MmPKCα protein or pre-treating cells with anti-PKCα antibodies (abs) failed to block viral entry (Fig. 3F through I). These results confirm MmPKCα acts as a signaling co-factor rather than a viral receptor, exerting its proviral effect via a post-attachment mechanism.

### MmPKCα facilitates RGNNV entry via MmMYL3-mediated macropinocytosis

**Fig 4:**
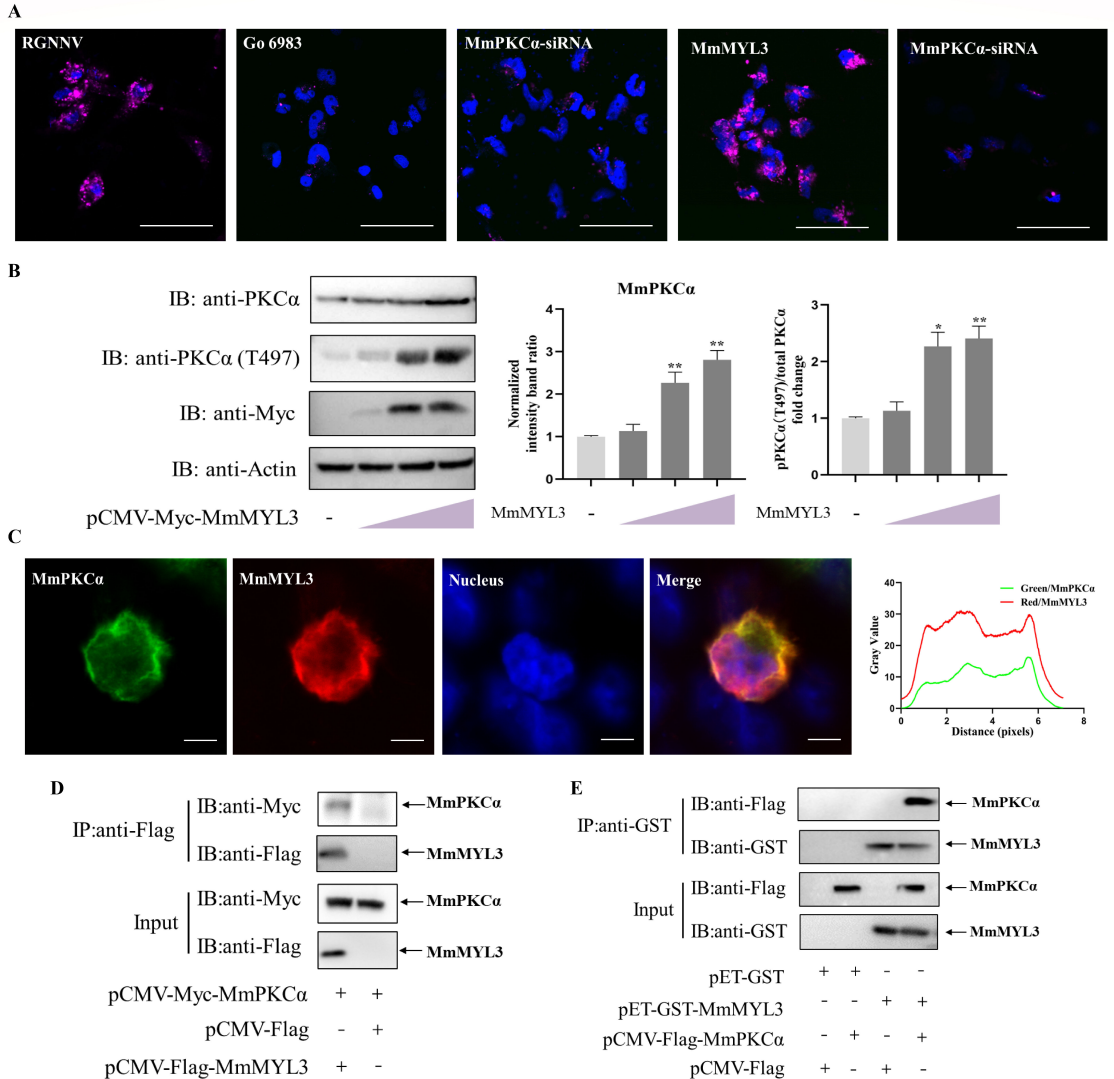
MmPKCα facilitates RGNNV entry via macropinocytosis. (**A**) hMMES1 or MmMYL3-overexpressing hMMES1 cells were treated with PBS or Go 6983 or transfected with siMmPKCα, and then infected with RGNNV (MOI = 1) in medium containing Alexa Fluor 647-conjugated dextran (10,000 MW). At 4 hpi, cells were fixed, and nuclei counterstained with DAPI and imaged on a confocal microscope. Bar = 10 µm. (**B**) hMMES1 cells were transfected with MmMYL3-Flag plasmids for 24 h and then were subjected to immunoblot assays using anti-PKCα or anti-PKCα (phosphor T497) abs. (**C**) MmPKCα-Flag and MmMYL3-Myc plasmids were transfected into HEK293T cells as indicated for immunofluorescence analysis by using anti-Flag (green) or anti-Myc (red) abs. Nuclei were stained with DAPI. Bar = 10 µm. (**D**) IP (with anti-Flag abs) and immunoblot analysis (with anti-Flag and anti-Myc abs) of HEK293T cells transfected with plasmids encoding MmMYL3-Flag and MmPKCα-Myc for 48 h. (**E**) The lysates of HEK293T cells transfected with the indicated plasmids were pulled down with purified MmMYL3-GST or GST proteins. The proteins bound to MmPKCα, and the inputs were immunoblotted with anti-GST and anti-Flag abs.The results are presented as mean ± SD. Statistical significance was determined by an unpaired two-tailed Student’s t test. *P < 0.05, **P < 0.01. Data are representative of three independent experiments.

Since MmPKCα is not a receptor but promotes RGNNV entry, we hypothesized that it acts downstream of the known RGNNV receptor MYL3, which mediates RGNNV entry via macropinocytosis. Using Alexa Fluor 647-conjugated dextran as a macropinocytosis tracer, we found that RGNNV infection robustly stimulated macropinocytosis in hMMES1 cells. RGNNV-induced dextran uptake in hMMES1 cells or MmMYL3-overexpressing hMMES1 cells was both effectively suppressed by either Go 6983 treatment or MmPKCα knockdown (Fig. 4A), demonstrating that MmPKCα is a necessary component of RGNNV-triggered macropinocytosis and MYL3’s pro-entry function is dependent on MmPKCα. We then probed the MmPKCα-MmMYL3 relationship. MmMYL3 overexpression increased both the total protein levels and Thr496 phosphorylation of MmPKCα (Fig. 4B), suggesting that MmMYL3 signaling activates MmPKCα. IF and Co-IP assays demonstrated an interaction between MmPKCα and MmMYL3 (Fig. 4C and D), which was confirmed by a GST pull-down assay (Fig. 4E). These data position MmPKCα as a critical downstream signaling effector of the MmMYL3 receptor, essential for executing the macropinocytic entry of RGNNV.

### MmPKCα inhibits cofilin phosphorylation via its CT domain to promote actin rearrangement

**Fig 5:**
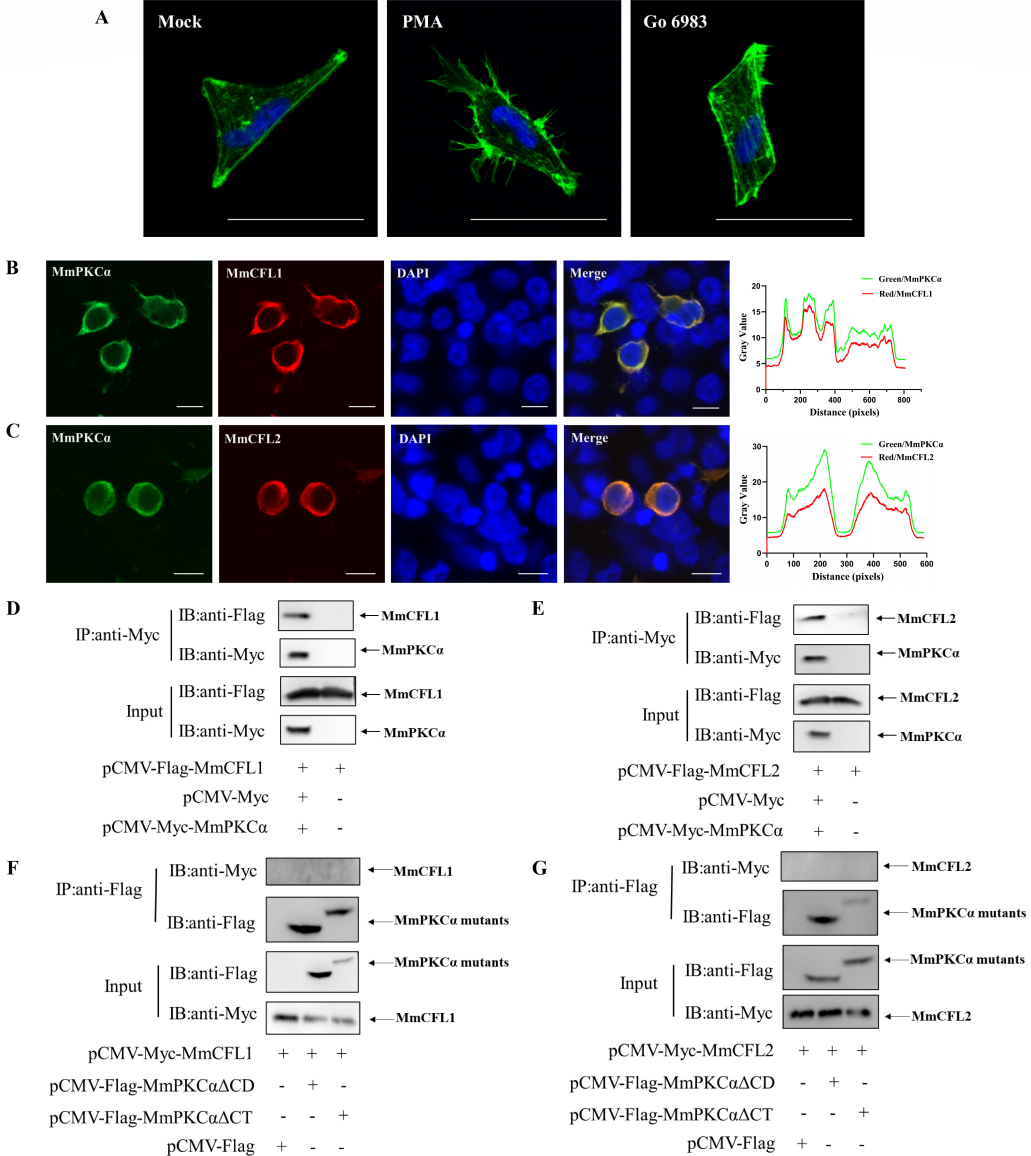
MmPKCα interacts with MmCFL1 and MmCFL2. (**A**) hMMES1 cells were exposed to PMA, Go6983, or PBS for 4 h, and actin filaments were labeled with iFluor 488 phalloidin (green), then fixed and permeabilized as described above. Nuclei were stained with DAPI. Images were captured with a ×100 oil immersion objective. Bar = 10 µm. (**B and C**) MmPKCα-Myc and MmCFL1-Flag or MmCFL2-Flag plasmids were transfected into HEK293T cells as indicated for immunofluorescence analysis by using anti-Myc (green) or anti-Flag (red) abs. Nuclei were stained with DAPI. Bar = 10 µm. (**D and E**) HEK293T cells were transfected with MmPKCα-Myc and MmCFL1-Flag or MmCFL2-Flag plasmids as indicated for 48 h, and the cell lysates were subjected to Co-IP analysis with anti-Myc magnetic beads as described above. (**F and G**) HEK293T cells were transfected with MmPKCα-ΔCD-Flag, MmPKCα-ΔCT-Flag, and MmCFL1-Myc or MmCFL2-Myc plasmids, respectively. At 48 h post-transfection, the cell lysates were subjected to Co-IP analysis with anti-Flag magnetic beads as indicated.

**Fig 6:**
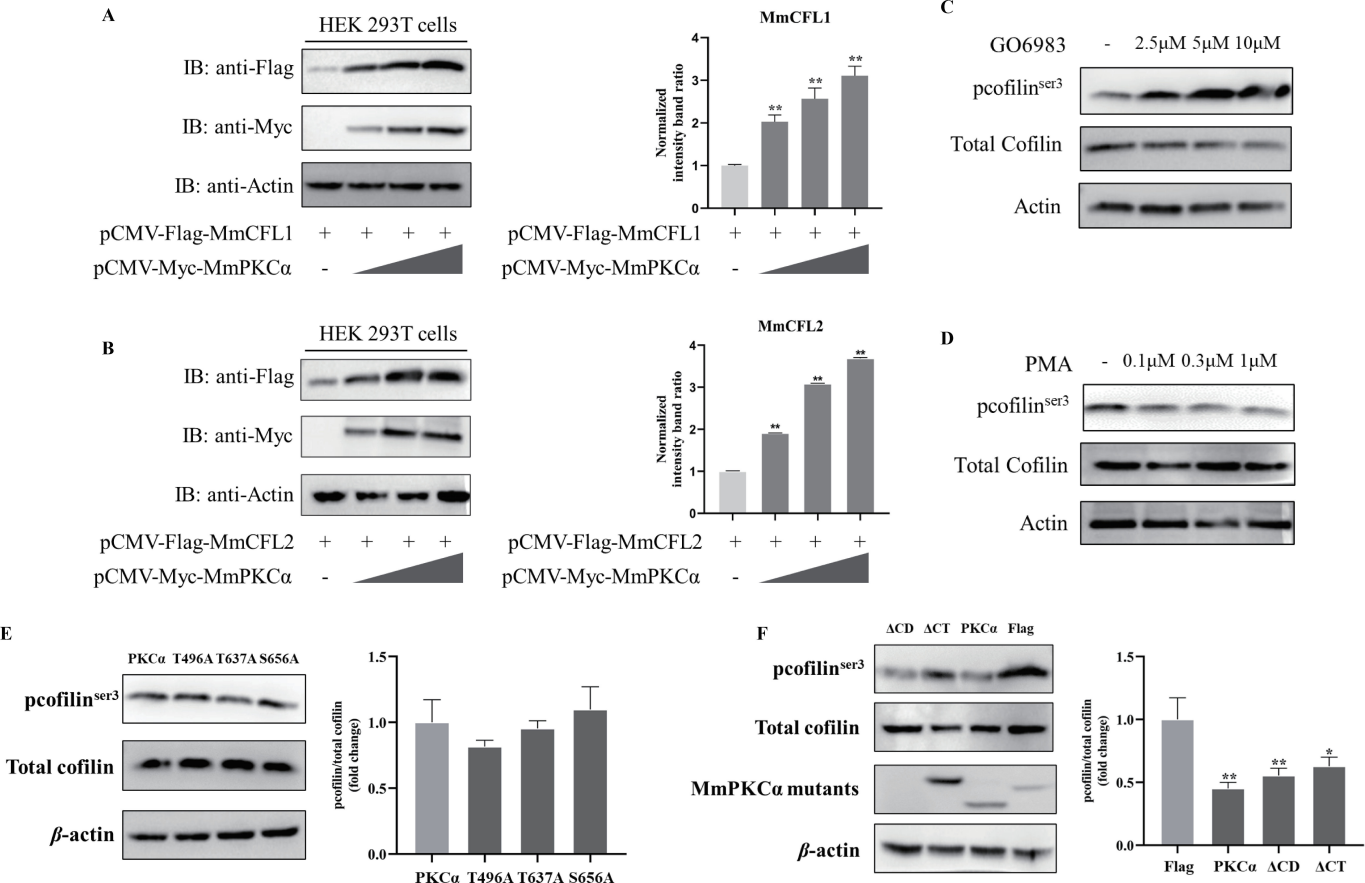
MmPKCα induces the dephosphorylation of cofilin via the CT domain. (**A and B**) HEK293T cells were transfected with the MmCFL1-Flag or MmCFL2-Flag and MmPKCα-Myc plasmids (0, 0.5, and 1 mg) for 24 h, and the cells were then lysed for immunoblot assays with the indicated abs. (**C and D**) hMMES1 cells were treated with Go 6983 or PMA for 4 h and then were subjected to immunoblot assays using anti-cofilin or anti-cofilin (phosphor S3) abs. (**E**) hMMES1 cells were transfected with MmPKCα-Flag or its point mutants of T496, T637, or S656 plasmids for 24 h and then were subjected to immunoblot assays using anti-cofilin or anti-cofilin (phosphor S3) abs. (**F**) hMMES cells were transfected with MmPKCα and its truncated mutant plasmids for 24 h and then were subjected to immunoblot assays using anti-cofilin or anti-cofilin (phosphor S3) abs. The results are presented as mean ± SD. Statistical significance was determined by an unpaired two-tailed Student’s t test. *P < 0.05, **P < 0.01. Data are representative of three independent experiments.

Macropinocytosis depends on actin cytoskeleton rearrangement, and cofilin is a key regulator of actin dynamics. We therefore investigated whether MmPKCα modulates cofilin activity. First, IF staining with iFluor-488-phalloidin showed that PMA treatment induced robust actin rearrangement in hMMES1 cells, while Go 6983 treatment had no effect (Fig. 5A), linking MmPKCα activation to actin dynamics. Next, we analyzed the interaction between MmPKCα and cofilin. MmPKCα colocalized with both MmCFL1 and MmCFL2 in HEK293T cells (Fig. 5B and C). Co-IP showed that MmPKCα interacted with MmCFL1/MmCFL2 via its CD and CT domains; deletion of either domain abolished binding (Fig. 5D through G). Furthermore, MmPKCα overexpression promoted the expression of both MmCFL1 and MmCFL2 in a dose-dependent manner in HEK293T cells (Fig. 6A and B). More importantly, functional assays showed that Go 6983 increased cofilin phosphorylation at Ser3 (inactive form), while PMA promoted cofilin dephosphorylation (active form) (Fig. 6C and D), indicating that MmPKCα suppresses cofilin Ser3 phosphorylation to maintain its actin-severing activity. Finally, we tested the role of MmPKCα domains in cofilin regulation. Mutation of MmPKCα’s phosphorylation sites (T496A/T637A/S656A) had no effect on cofilin Ser3 phosphorylation (Fig. 6E). Deletion of the MmPKCα CT domain, but not the CD domain, abolished its ability to inhibit cofilin phosphorylation (Fig. 6F). These results demonstrate that the MmPKCα CT domain is critical for binding cofilin and suppressing its Ser3 phosphorylation, thereby promoting actin rearrangement required for macropinocytosis.

## DISCUSSION

**Fig 7:**
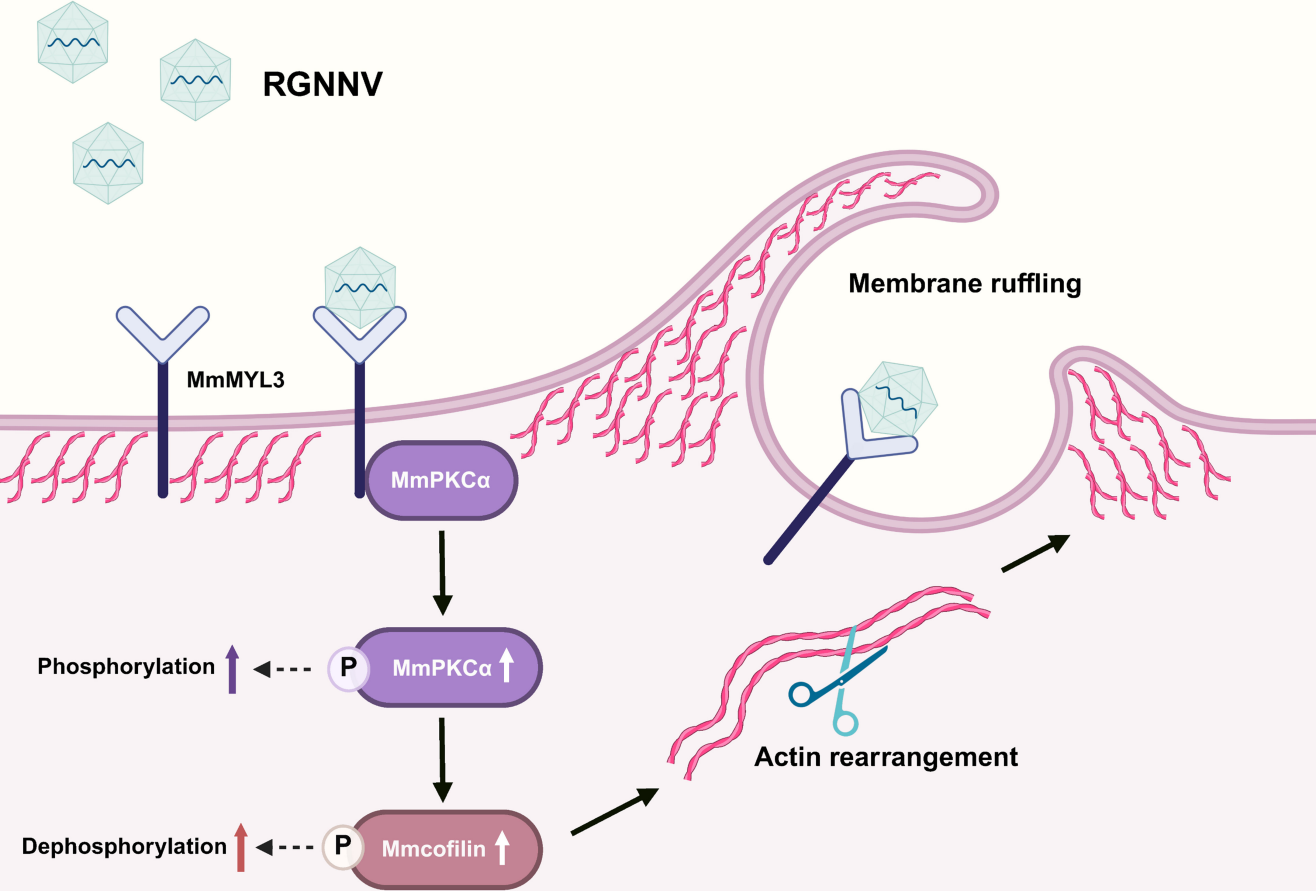
Model of RGNNV entry into host cells via macropinocytosis mediated by MmMYL3-MmPKCα-cofilin signaling pathway. RGNNV particles engage hMMES1 cells through interactions between MmMYL3 (a NNV receptor on the cell surface) and CP, which is followed by interactions between MmMYL3 and MmPKCα. Then, MmPKCα phosphorylation is stimulated by MmMYL3, which in turn induces cofilin dephosphorylation essential for actin rearrangement. This dynamic peripheral actin rearrangement leads to membrane ruffle formation and macropinocytosis.

Viral entry is a critical determinant of infection success, and understanding the host signaling pathways hijacked by viruses provides key insights into pathogenesis and therapeutic targeting. In this study, we delineate a novel signaling axis that RGNNV exploits to invade host cells. Our data support a model in which RGNNV, via its CP, engages the MmMYL3 receptor, leading to the recruitment and activation of MmPKCα. Activated MmPKCα then directly interacts with and promotes the dephosphorylation of cofilin, culminating in the actin-driven membrane ruffling and macropinocytosis that propel RGNNV into the host cell (Fig. 7). This cascade clarifies the molecular mechanisms underlying RGNNV entry and highlights PKCα functioning as a critical link between the cell surface receptor MmMYL3 and the intracellular actin-remodeling machinery in teleost.

PKCα is a member of classical PKC with ubiquitous expression and different cellular localization, sitting at the crossroads of many signal transduction pathways and is implicated in a wide range of cellular responses, including cell proliferation, differentiation, migration, and apoptosis (25, 26). The role of PKCα in viral entry has been documented for several mammalian viruses (10, 11, 16), but its function in teleost viruses has remained enigmatic. We found that RGNNV actively upregulates and phosphorylates MmPKCα, and its kinase activity is indispensable for efficient viral entry. Notably, MmPKCα directly interacts with RGNNV CP via its CT domain; the interaction is independent of PKCα phosphorylation status. This viral-host protein interaction likely contributes to MmPKCα activation, as CP overexpression alone was sufficient to induce MmPKCα phosphorylation. However, despite MmPKCα localizing to the cell surface and interacting with MmMYL3, comprehensive receptor validation assays, including unaltered viral binding, inability to render non-susceptible cells permissive, and failure of neutralization by abs or recombinant protein, conclusively demonstrated that MmPKCα is a signaling co-factor rather than a binding receptor, explaining its ability to promote entry without affecting initial viral attachment. This places MmPKCα in a growing category of host factors that NNV co-opt for post-entry steps after engaging its primary receptor.

Macropinocytosis is a distinct endocytosis pathway that is associated with considerable cell-wide plasma membrane ruffling induced by a virus, and this process depends on the cooperative action of multiple kinases (7). Previous studies have shown that the inhibitors of PKC can reduce the macropinocytosis caused by viral infection, such as foot-and-mouth disease virus, infectious pancreatic necrosis virus, and severe acute respiratory syndrome coronavirus 2 (27–29). Our findings revealed that both pharmacological inhibition of PKC and knockdown of MmPKCα effectively attenuated RGNNV-induced macropinocytosis. We previously identified MmMYL3 as an RGNNV receptor that mediates entry exclusively via macropinocytosis (4), but the signaling events linking MmMYL3 to macropinocytic machinery remained unclear. Here, we show that MmMYL3 directly interacts with MmPKCα and activates it via Thr496 phosphorylation, and that MmMYL3’s ability to enhance RGNNV entry is dependent on PKC activity. These findings establish MmPKCα as a critical downstream effector of MYL3, bridging receptor engagement to the execution of macropinocytosis. This is consistent with mammalian studies showing that PKC family members act downstream of cell surface receptors to regulate endocytosis (30). The fact that PKC inhibitors significantly block MmMYL3’s ability to enhance infection underscores that the proviral function of this receptor is channeled through PKCα signaling. This positions the MmMYL3-MmPKCα axis as a crucial signaling module for initiating NNV macropinocytosis. In macropinocytosis, PKC family normally plays a central role in activating key regulators of cytoskeletal rearrangement, including cofilin and Rab GTPases (7). In contrast, while CME also involves alterations in cell membrane morphology, it mainly depends on specific structural proteins, such as clathrin. Moreover, the interaction between MmPKCα and MmMYL3 further addresses the specific role of MmPKCα in NNV-induced macropinocytosis.

Actin cytoskeleton rearrangement is the mechanical driver of macropinocytosis, and cofilin is a master regulator of this process (31). Actin binding activity of cofilin is controlled via phosphorylation (inactivation) and dephosphorylation (activation) of a conserved serine residue at Ser3, and PKCs have been recognized as potent regulators to affect this process (32, 33). Moreover, the PKCδ-cofilin signaling pathway plays a regulatory role in virus-induced macropinocytosis (24, 34). To explore whether MmPKCα shares functional similarities with cofilin, we first confirmed the interaction between MmPKCα and both MmCFL1 and MmCFL2. PKCα contains a regulatory domain that maintains the enzyme in an inactive conformation, and the catalytic domain is able to phosphorylate the appropriate substrate for further downstream processing (35). Our findings revealed that the deletion of CD and CT domains belonging to the MmPKCα catalytic domain abolished its ability to interact with MmCFL1 and MmCFL2, indicating that CD and CT domains are essential to the interaction between MmPKCα and cofilin. In addition to the interaction between MmPKCα and cofilin, our results showed that MmPKCα enhanced the expression of both exogenous and endogenous MmCFL1 and MmCFL2 and simultaneously suppressed cofilin phosphorylation, thereby promoting the actin-severing activity of cofilin. Notably, our findings revealed that the mutation of T497, T637, and S656 in MmPKCα did not significantly induce the dephosphorylation of cofilin at Ser3. However, the deletion of the CT domain markedly inhibited cofilin dephosphorylation. Considering T637 and S656 are both located within the CT domain, these results suggested that MmPKCα-mediated regulation of cofilin activity might rely on the CT domain itself or the synergistic phosphorylation of T637 and S656. In mammalian systems, cofilin Ser3 phosphorylation is tightly controlled by a balance of kinases (LIM-kinase [LIMK], testicular protein kinases [TESK]) and phosphatases (slingshot protein phosphatases [SSH]) (36–38). Extending this conserved regulatory framework to teleost, we propose two non-mutually exclusive models for MmPKCα CT domain function: First, the CT domain may directly interact with SSH or its upstream regulators, facilitating SSH recruitment to cofilin and enhancing its dephosphorylation. Second, the CT domain could sterically block LIMK or TESK from accessing cofilin, thereby reducing Ser3 phosphorylation. Future studies will validate these hypotheses by investigating the interaction between MmPKCα CT domain and key components of the cofilin phosphorylation/dephosphorylation machinery in marine medaka.

Notably, current PKC inhibitors lack subtype specificity, restricting their *in vivo* applicability and therapeutic potential. Our study identifies the MmPKCα CT domain as a dual-function module: it mediates interactions with RGNNV CP and regulates cofilin phosphorylation. This dual role makes the CT domain a promising target for developing subtype-specific inhibitors. Unlike broad-spectrum PKC inhibitors, CT domain-targeted agents would selectively disrupt MmPKCα-dependent RGNNV entry without interfering with other PKC isoforms or host endocytic homeostasis.

In this study, we show that MmPKCα promotes RGNNV entry via the novel MmMYL3-MmPKCα-cofilin signaling axis. As a key downstream effector of the RGNNV receptor MmMYL3, MmPKCα facilitates MmMYL3-mediated macropinocytosis by binding cofilin (MmCFL1/2) and suppressing its Ser3 phosphorylation to drive actin rearrangement. Our findings identify MmPKCα as a pivotal signaling hub in RGNNV infection and a potential therapeutic target for anti-VNN strategies.

## MATERIALS AND METHODS

### Cells, virus, and compounds

hMMES1 cells were cultivated in ESM4 medium at 28°C as previously described (39). HEK293T cells were cultured in DMEM medium supplemented with 10% heat-inactivated fetal bovine serum (FBS, Life-iLab, China) under standard conditions of 37°C with 5% CO_2_.

The RGNNV strain (isolated from diseased sea perch larvae in Guangdong Province, China) was propagated in LJB cells and stored at −80°C as viral stocks (40).

A suite of abs was employed for various assays: anti-Flag (M20008), anti-Myc (M20002), anti-Actin (M20011), and anti-GST abs (MA9024) were purchased from Abmart (Guangzhou, China), while anti-PKCα (ab32376), anti-PKC (phospho T497) (ab59411), anti-cofilin (ab124979), and anti-cofilin (phospho S3) (ab283500) abs were purchased from Abcam (Cambridge, England). Secondary abs, including goat anti-rabbit IgG-HRP and anti-mouse IgG-HRP, and phenylmethylsulfonyl fluoride, were purchased from Beyotime (Shanghai, China). Additionally, donkey anti-mouse or goat anti-rabbit IgG (H+L) highly cross-adsorbed secondary abs, as well as Alexa Fluor 555 and Alexa Fluor 488, Alexa Fluor 647-dextran (10,000 MW), were obtained from Invitrogen (Carlsbad, CA, USA). iFluorTM 488 (40736ES75) phalloidin was purchased from Yeasen, while magnetic beads of anti-Flag (HY-K0207), anti-c-Myc (HY-K0206), and anti-GST (HY-K0222) were purchased from MedChemExpress (Monmouth Junction, NJ, USA). Essential chemicals, such as 4′,6-diamidino-2-phenylindole (DAPI) (D9542), isopropyl-1-thio-β-d-galactopyranoside (IPTG) (I6758), and Triton X-100 (93443), were procured from Sigma-Aldrich (St. Louis, MO). Specific inhibitors, including PMA (TQ0198), Go 6983 (T6313), sotrastaurin (T6278), IPA-3 (T6546), and LY294002 (T2008), were purchased from TargetMol (Shanghai, China).

### Plasmid construction

The open reading frame of MmPKCα (accession number: XM_036209694), MmMYL3 (accession number: XM_024288939.2), MmCFL1 (accession number: XM_024283295.2), MmCFL2 (accession number: XM_024267965.2), and MmPKCα deletion mutants were amplified by PCR and cloned into the *pCMV-Flag* or *pCMV-Myc* vector. All plasmids were confirmed by DNA sequencing. Primers used for plasmid construction are listed in Table S1 in the supplemental material. Myc-tagged and Flag-tagged CP and its deletion mutants were constructed as previously described (41).

### Cell transfection and RGNNV infection

hMMES1 in six-well plates (1 × 10^6^ cells/well) were transfected with different plasmids using Lipofectamine 8000 (Beyotime, China) following the manufacturer’s instructions. At 24 h post-transfection, the cells were infected with RGNNV at a multiplicity of infection (MOI) of 1 for the indicated times and examined by qRT-PCR.

### Quantitative reverse transcription-PCR

Total RNA of cultured cells was extracted with TRIzol reagent (Invitrogen, CA, USA) according to the manufacturer’s instructions and was further reverse transcribed into cDNA through the GoScript Reverse Transcription Mix (Promega, Madison, USA). A LightCycler 480 II (Roche Applied Science, Germany) and qPCR SYBR Green Master Mix (Yeasen, China) were used for qRT-PCR analysis by using gene-specific primers (Table S1). mRNA relative expression levels were evaluated from triplicate experiments and normalized to marine medaka *β-actin*. The relative fold induction of genes was calculated using the threshold cycle (2^−ΔΔCT^) method and presented as mean ± standard deviation (SD).

### RNA interference

siRNAs targeting MmPKCα (siMmPKCα) were synthesized by the Ribobio company (Guangzhou, China), including siRNA-1 (5′-CTTGCGACATGAACGTTCA-3′), siRNA-2 (5′-CCAGATGGAACGAGCACTT-3′), siRNA-3 (5′-GCCATGAATTTGTCACGTT-3′), and control siRNA (NC) (5′-UUCUCCGAACGUGUCACGUTT-3′). Transfection of siRNAs was conducted using a mixture of three different siRNAs at concentrations of 50 or 100 nM as described previously (42).

### Western blot and Co-IP

The cells were lysed with lysate buffer (Beyotime, China) and boiled for 10 min with 1% SDS for SDS-PAGE separation. The proteins were transferred onto polyvinylidene difluoride membranes (Millipore, USA) and then blocked with 5% nonfat dried milk for 1 h at room temperature (RT), followed by incubation with primary abs at 4°C overnight, including anti-Flag (1:4,000), anti-Myc (1:4,000), anti-PKCα (1:1,000), anti-CP (1:1,000), anti-cofilin (1:1,000), anti-cofilin (phosphor S3) (1:1,000), and anti-actin (1:4,000) abs. Subsequently, membranes were further probed with goat anti-rabbit or anti-mouse IgG (H+L) highly cross-adsorbed secondary abs (1:1,000) for 1 h at RT and analyzed using enhanced chemiluminescence immunoblotting detection reagents (Millipore, USA) on a chemiluminescence instrument (Sage Creation, China).

For the Co-IP assay, cell extracts were incubated with anti-Flag/Myc magnetic beads at 4°C overnight. After incubation, the beads were washed five times with lysis buffer and eluted with 1% SDS buffer for boiling. The eluted samples were subjected to western blot analysis.

### Protein purification and pull-down assays

The *pET-GST-MmPKCα*, *pET-GST-MmMYL3,* and empty *pET-GST* were transformed into *Escherichia coli* BL21 (DE3) separately. Bacteria were grown to an OD_600_ of 0.8–1.0 and then induced with 0.5 mM IPTG for 12 h at 20°C in a shaking incubator. GST-tagged proteins were purified using the GST spin purification kit (Beyotime, China) according to the manufacturer’s instructions. Briefly, the bacterial solution was collected and washed three times with PBS. Lysozyme was added to a final concentration of 1 mg/mL and placed on ice for 30 min, then centrifuged at 4°C for 10 min. The supernatant was purified using the BeyoGold GST-tag Purification Resin as described previously (43). Finally, the protein was eluted from the column. The purified protein was stained with Coomassie brilliant blue.

GST-Tag magnetic beads were first mixed with the GST-fused MmPKCα protein for 4 h at RT. Then, the beads were incubated with protein lysates from HEK293T cells transfected with *pCMV-Flag-MmPKCα* or *pCMV-Flag* vector at 4°C overnight and finally analyzed by western blot.

### Blocking assays

MmPKCα-GST protein was affinity-purified as described above. RGNNV was incubated with different concentrations (100 or 500 ng) of recombinant MmPKCα-GST protein for 4 h at 4°C. Then, hMMES1 cells were incubated with virus and protein mixtures for 4 h at 4°C. Similarly, cells were treated with RGNNV preincubated with BSA (500 ng) as a control. Then, cells were harvested, and *CP* mRNA was measured as described above.

Due to the unavailability of anti-MmPKCα abs and the high homogeneity of PKCα at the N-terminus domain between human and fish, cells were incubated with anti-human PKCα abs (1:50) for 4 h at 28°C. After washing with fresh media, cells were infected with RGNNV at 4°C for 2 and 4 h, respectively. As a control, hMMES1 cells were in parallel pretreated with normal rabbit IgG. Cells were then washed three times with PBS to remove free virus particles and harvested for total RNA extraction and qRT-PCR detection of *CP*.

### IF assays

For assessment of the colocalization, HEK293T cells plated on coverslips in 24-well plates were transfected with different plasmids. After transfection for 24 h, cells were washed with PBS and fixed with 4% paraformaldehyde for 10 min at RT. Cells were treated with 0.1% Triton X-100 for membrane permeabilization. After being washed three times with PBS, cells were blocked with PBS containing 5% bovine serum albumin at RT for 1 h and were incubated with primary abs at 4°C overnight, including anti-Flag (1:400) and anti-Myc (1:400) abs, followed by incubation with Alexa Fluor 555- or 488-conjugated secondary abs against mouse or rabbit IgG (1:400). Cells were then washed with PBS and stained the cell nuclei with DAPI for 10 min. The coverslips were washed with PBS and observed under an SP8 Leica laser confocal microscopy imaging system (SP8, Leica, Germany).

For the dextran uptake assay for macropinocytosis, hMMES1 cells on coverslips were treated with the indicated chemical compound following a published procedure (44). After treatment, the cells were serum-starved for 16 h. The cells were infected with RGNNV in media containing Alexa Fluor 647-conjugated dextran (10,000 MW), incubated for 4 h at 28°C, and washed and fixed for 16 h with 4% PFA at 4°C. Samples were viewed and evaluated by confocal microscopy.

To observe actin cytoskeleton dynamics after being treated with PMA or Go 6983, hMMES1 cells were exposed to different compounds and incubated for 4 h. The temperature shift was performed after washing with PBS by using prewarmed (28°C) serum-free medium for 30 min. Afterward, cells were fixed and permeabilized as described above. iFluor 488 phalloidin and DAPI were used to stain actin filaments and nuclei of cells, respectively.

### Inhibitor treatment assays

Cells were treated with various inhibitors to investigate the impact of various kinases on the role of MmMYL3 in facilitating the entry of RGNNV. MmMYL3-overexpressing hMMES1 cells were treated with inhibitors of kinases for 4 h, then infected with RGNNV for 4 h at 28°C. Next, the cells were washed with PBS three times to remove any unbound viruses. DMSO was used as a control. Total RNA of cells was extracted for qRT-PCR detection.

hMMES1 cells were treated with various inhibitors to investigate the effect of MmPKCα on RGNNV entry or cofilin phosphorylation. PMA or Go 6983 was added when the cell density reached approximately 85%. After the cells were incubated with small molecule compounds of different concentrations for 4 h, the culture supernatant was discarded, and the new medium was added for 24 and 48 h, respectively. DMSO was used as a control. Total RNA of cells was extracted for qRT-PCR detection. Cell lysate was used for western blot.

### Statistical analysis

Data were collected from three independent experiments, analyzed with SPSS version 20.0, and presented as the means ± SD. Student’s t test or one-way analysis of variance was used for the statistical comparisons between two groups or multiple groups, respectively. *P* value <0.05 was considered to indicate a statistically significant difference; *P* value <0.01 was considered to indicate a highly significant difference.

## Data Availability

All data supporting the findings of this study are available within the article. Further details can be obtained from the corresponding author.

## SUPPLEMENTAL MATERIAL

Supplemental material jvi.02064-25-s0001.docx Figures S1 and S2; Table S1.

ASM does not own the copyrights to Supplemental Material that may be linked to, or accessed through, an article. The authors have granted ASM a non-exclusive, world-wide license to publish the Supplemental Material files. Please contact the corresponding author directly for reuse.
