# Supplementary material for: Marine medaka PKCα promotes red-spotted grouper nervous necrosis virus entry by orchestrating MYL3-mediated macropinocytosis and cofilin-dependent actin remodeling
Source: J Virol. 2026 Feb 25;100(3):e02064-25. doi: 10.1128/jvi.02064-25 (PMC13011413; doi:10.1128/jvi.02064-25)
Supplement: Supplemental material — Figures S1 and S2; Table S1. [file jvi.02064-25-s0001.docx]

**Supplemental Figures and Table**

**
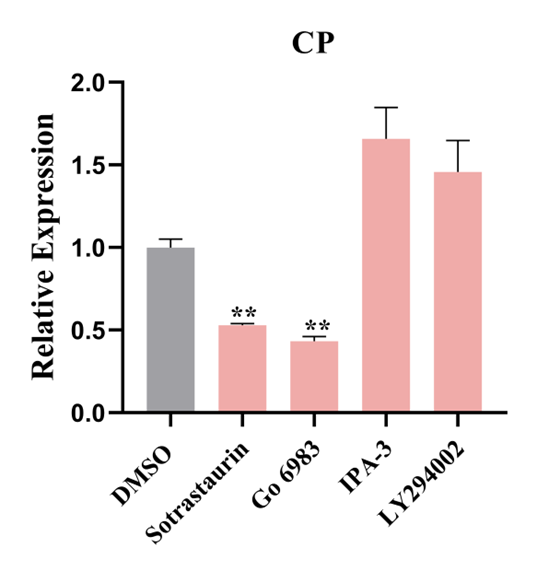
**

**Fig S1. The PKC inhibitors block MYL3-mediated promotion of RGNNV entry.** MmPKCα-overexpressing hMMES1 cells were treated with different kinase inhibitors and incubated with RGNNV for 4 h at 28°C, and the expression of *CP* was analyzed by quantitative reverse transcription PCR (qRT-PCR). The results are presented as mean ± SD. Statistical significance was determined by an unpaired two-tailed Student’s t test. **P* < 0.05, ***P* < 0.01. Data are representative of three independent experiments.


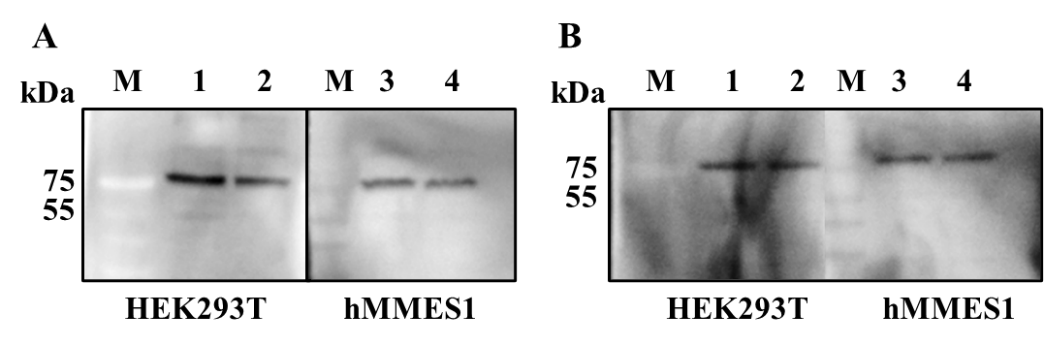


**Fig S2. Validation of PKCα and its phospho-T497 antibodies.** (A) Validation of the PKCα antibody. Lane M, marker; Lane 1, HEK293T cell lysate; Lane 2, HEK293T cell lysate overexpressing MmPKCα; Lane 3, hMMES1 cell lysate; Lane 4, hMMES1 cell lysate overexpressing MmPKCα. (B) Validation of the PKCα (phospho-T497) antibody. Lane M, marker; Lane 1, HEK293T cell lysate; Lane 2, HEK293T cell lysate overexpressing MmPKCα; Lane 3, hMMES1 cell lysate; Lane 4, hMMES1 cell lysate overexpressing MmPKCα.

**Table S1. Primer sequences used by application.**

| **qRT-PCR Primers** | | |
| --- | --- | --- |
| Genes | 5’ Primer | 3’ Primer |
| *MmPKCα* | AACAGGTTCGCCCGCAAAGGT | CAGCAAACTTGGCACTGAAATCCTT |
| *CP* | GTCGGCTGATACTCCTGTGTG | CTCCAGTTCCAAGGCTGTAGT |
| *RDRP* | GCTTTATGCGTGAGTGCGTC | GCTGTTTCCGTCTGTTGTGAG |
| *β-actin* | TTCAACAGCCCTGCCATGTA | CCTCCAATCCAGACAGAGTATT |
| *MmMYL3* | ACGCCTTCCTGCCCATGCTG | CTGCCGTTCTCGTCCTCCTG |
| *18S rRNA* | CAGCCACCCGAGATTGAGCA | TAGTAGCGACGGGCGGGTGT |
| **Plasmid Construction Primers** | | |
| pCMV-Flag/Myc-MmPKCα | GGAATTCGGATGGCTGATGGAAGTTTCAACGAGT | GGGGTACCTTATACACTGTTGACAAGTGAGGGG |
| pCMV-Flag-MmPKCα-T496A | ACAACCAGAgCCTTCTGCGGAACCCCAGACTA | CAGAAGGcTCTGGTTGTCACATTTTCTGACATG |
| pCMV-Flag-MmPKCα-T637A | CTCAGCTCgCCCCACCAGACGAGCTGGTCATC | TGGTGGGGcGAGCTGAGGCTGGGTGCGTGTGA |
| pCMV-Flag-MmPKCα-T656A | CAGGGTTCgCTTTCATCAACCCCGAGTTTATG | GATGAAAGcGAACCCTGCAAACTCTGCTTGGT |
| pCMV-Flag- MmPKCα-ΔC1A (1-34aa) | GGAATTCGG ATGGCTGATGGAAGTTTCAACGAGT | GTTCTTCACTTCGTGGACGTTTTTT |
| pCMV-Flag- MmPKCα-ΔC1A (85-671aa) | ACGTCCACGAAGTGAAGAAC CCCGGGGCGGATAAGGG | GGGGTACC TTATACACTGTTGACAAGTGAGGGG |
| pCMV-Flag- MmPKCα-ΔC1B (1-99aa) | GGAATTCGG ATGGCTGATGGAAGTTTCAACGAGT | CTTTGTTCTGGGATCGTCAGTGTCA |
| pCMV-Flag- MmPKCα-ΔC1B (150-671aa) | CTGACGATCCCAGAACAAAG GGAACGGATCACACTGAGAG | GGGGTACC TTATACACTGTTGACAAGTGAGGGG |
| pCMV-Flag- MmPKCα-ΔC2 (1-169aa) | GGAATTCGG ATGGCTGATGGAAGTTTCAACGAGT | GTCCCCACTGACCTCACACCTGAGG |
| pCMV-Flag- MmPKCα-ΔC2 (274-671aa) | TCAGGTGTGAGGTCAGTGGGGAC AAGCTGTTGTGCCAGGAGGAGGGGG | GGGGTACC TTATACACTGTTGACAAGTGAGGGG |
| pCMV-Flag- MmPKCα-ΔCD (1-337aa) | GGAATTCGG ATGGCTGATGGAAGTTTCAACGAGT | GTCATTGAGTTTGACCTGGCTCAGG |
| pCMV-Flag- MmPKCα-ΔCD (597-671aa) | CCTGAGCCAGGTCAAACTCAATGAC AGGCGGATCGACTGGGACCGCCTCC | GGGGTACC TTATACACTGTTGACAAGTGAGGGG |
| pCMV-Flag- MmPKCα-ΔCT (1-596aa) | GGAATTCGG ATGGCTGATGGAAGTTTCAACGAGT | GAAGAAAGCATGCTCCCTGATGTCC |
| pET-GST- MmPKCα | CCGGAATTC ATGGCTGATGGAAG | CCGCTCGAG TTATACACTGTTGAC |
| pCMV-Flag/Myc-CP | GGAATTCGGGTACGCAAAGGTGAGAAGAAAT | ACGCGTCGACTTAGTTTCCCGAGTCAACCCTG |
| pCMV-Myc/Flag-CP-ΔARM-(34-338aa) | GGAATTCGGCGCACTGACGCACCTGTGTCTA | ACGCGTCGACTTAGTTTCCCGAGTCAACCCTG |
| pCMV-Myc/Flag-CP-Δarm-(1-33aa) | GGAATTCGGGTACGCAAAGGTGAGAAGAAAT | ATTACTACGCCGACGATTGTT |
| pCMV-Myc/Flag-CP-Δarm-(52-338aa) | AACAATCGTCGGCGTAGTAATACCAATGACGTCCATCTCTCA | ACGCGTCGACTTAGTTTCCCGAGTCAACCCTG |
| pCMV-Myc/Flag-CP-ΔLR-(1-213aa) | GGAATTCGGGTACGCAAAGGTGAGAAGAAAT | ACGCGTCGACTTGAGCGTTCCATCTCTTGAG |
| pCMV-Myc/Flag-CP-ΔLR-(221-338aa) | GTTCCATCTCTTGAGCCCATCATGACACAAGG | ACGCGTCGACTTAGTTTCCCGAGTCAACCCTG |
| pCMV-Myc/Flag-CP-ΔP-(1-220aa) | GGAATTCGGGTACGCAAAGGTGAGAAGAAAT | GGAATTCCCATCATGACACAAGGTTCC |
| pCMV-Myc/Flag-CP-ΔS-(1-51aa) | GGAATTCGGGTACGCAAAGGTGAGAAGAAAT | ACGCGTCGACTGTAACTGGATTTGGACGTGGG |
| pCMV-Myc/Flag-CP-ΔS-(214-338aa) | GATTTGGACGTGGGACACCTGAAGAGACTACCGCT | ACGCGTCGACTTAGTTTCCCGAGTCAACCCTG |
| pCMV-Flag/Myc-MmCFL1 | CGGAATTCGGATGGCCTCTGGCGTTAAAGTC | GGGGTACCTTATAAAGGGGATCCTTCC |
| pCMV-Flag/Myc-MmCFL2 | CGGAATTCGGATGGCATCAGGTGTGACAG | GGGGTACCTCACAGCGGCTTGCCTTCCAG |
| pCMV-Flag/Myc-MmMYL3 | ACGCCTTCCTGCCCATGCTG | CTGCCGTTCTCGTCCTCCTG |
| pET-GST- MmMYL3 | CCGGAATTC ATGACCGAGTTCAC | CCGCTCGAGTTACACAGACATG |
